# Development of an inducible transposon system for efficient random mutagenesis in *Clostridium acetobutylicum*

**DOI:** 10.1093/femsle/fnw065

**Published:** 2016-03-20

**Authors:** Ying Zhang, Shu Xu, Changsheng Chai, Sheng Yang, Weihong Jiang, Nigel P. Minton, Yang Gu

**Affiliations:** 1 Clostridia Research Group, BBSRC/EPSRC Synthetic Biology Research Centre (SBRC), School of Life Sciences, University of Nottingham, Nottingham NG7 2RD, UK; 2 Key Laboratory of Synthetic Biology, Institute of Plant Physiology and Ecology, Shanghai Institutes for Biological Sciences, Chinese Academy of Sciences, Shanghai 200032, China; 3 Jiangsu National Synergetic Innovation Center for Advanced Materials, SICAM, 200 North Zhongshan Road, Nanjing 210009, China; 4 Shanghai Collaborative Innovation Center for Biomanufacturing Technology, 130 Meilong Road, Shanghai 200237, China

**Keywords:** *Clostridium*, transposon, random mutagenesis, xylose-inducible promoters

## Abstract

*Clostridium acetobutylicum* is an industrially important Gram-positive organism, which is capable of producing economically important chemicals in the ABE (Acetone, Butanol and Ethanol) fermentation process. Renewed interests in the ABE process necessitate the availability of additional genetics tools to facilitate the derivation of a greater understanding of the underlying metabolic and regulatory control processes in operation through forward genetic strategies. In this study, a xylose inducible, *mariner*-based, transposon system was developed and shown to allow high-efficient random mutagenesis in the model strain ATCC 824. Of the thiamphenicol resistant colonies obtained, 91.9% were shown to be due to successful transposition of the *catP-*based mini-transposon element. Phenotypic screening of 200 transposon clones revealed a sporulation-defective clone with an insertion in *spo0A*, thereby demonstrating that this inducible transposon system can be used for forward genetic studies in *C. acetobutylicum*.

## Abstract (precis)

A useful method for forward genetic studies in *Clostridium acetobutylicum*.

## INTRODUCTION

Heightened concerns over global warming and fossil fuel supply, security and prices have led to a resurgence of interest in the sustainable production of chemicals and fuels. In this regard, saccharolytic *Clostridium* species are of particular interest given their former use on a commercial scale in the so-called Acetone-Butanol-Ethanol (ABE) fermentation process, producing the solvents acetone, butanol and ethanol. While several different saccharolytic *Clostridium* species have been deployed in the ABE process, *C. acetobutylicum* is widely regarded as the model organism. Not surprisingly, therefore, the genome of *C. acetobutylicum* ATCC 824 was the first clostridial genome sequence to be described (Nolling *et al*. 2001).

Renewed interest in reviving the ABE process has led to concerted efforts to derive genetic tools that may be deployed to both better understand the underlying metabolic and regulatory control processes, and to bring about improvements in productivity. Accordingly, genetic tools for directed gene disruption have been widely described. These include gene knock-down methods based on antisense RNA (Desai and Papoutsakis 1999; Tummala, Junne and Papoutsakis 2003), insertional gene disruption methods based on the bacterial mobile group II intron from the *ltrB* gene of *Lactococcus lactis* (Heap *et al*. 2007, 2010a,b; Shao *et al*. 2007), gene knock-out methods reliant on homologous recombination and the use of replicative and non-replicative vectors (Al-Hinai, Fast and Papoutsakis 2012; Heap *et al*. 2012; Leang *et al*. 2013; Ehsaan *et al*. 2016), and recently developed CRISPR-Cas9 system (Wang *et al*. 2015; Xu *et al*. 2015). Such tools have provided the basis for reverse genetic strategies that have focused on the key solvent producing pathways and have led to a number of successful rational metabolic engineering strategies for altering solvent productivity in *C. acetobutylicum* (Scotcher, Rudolph and Bennett 2005; Jang *et al*. 2012).

By way of contrast, scant attention has been paid to forward genetic approaches reliant on random mutagens typified by transposon elements. Two conjugative transposons Tn*916* and Tn*1514* have been studied in *C. acetobutylicum* ATCC 824, *C. saccharobutylicum* P262 and *C. beijerinckii* NCIMB 8052 (Woolley *et al*. 1989; Bertram, Kuhn and Durre 1990; Mattsson and Rogers 1994). These were used to isolate a number of interesting mutants, including one deficient in ‘degeneration’ (the loss in prolonged culture of the ability to produce solvents) (Kashket and Cao 1993) and others exhibiting increased butanol tolerance (Liyanage, Young and Kashket 2000). However, a number of factors mean these tools are far from ideal. Principles among these are their large size and low efficiency of transposition, in the case of Tn*916* the existence of ‘hot spots’ in the chromosome where the majority of the insertions locate and, in the case of Tn*1545*, the predilection to insert in multiple copies (probably attributed to a high rate of vector retention). This latter property significantly complicates the association of genotype with phenotype, hence, requiring further experimentation to understand the basis of the observed phenotypes.

A number of non-conjugative transposon mutagenesis systems have been described for pathogenic clostridia recently, including two *mariner*-based transposon systems, in *C. perfringens* (Liu *et al*. 2013) and *C. difficile* (Cartman and Minton 2010). The later was recently adapted to be used in *C. acetobutylicum* ATCC 824, by equipping the host cell with the ability to produce a foreign sigma factor, TcdR (Zhang, Grosse-Honebrink and Minton 2015). Although the new system proved to be highly effective, it is reliant on prior addition of the TcdR-encoding gene and is, therefore, not applicable if a wild-type clostridial host is the desired target. In addition, embracing the fast developed next generation DNA sequencing technology, transposon-directed insertion site sequencing was recently used in *C. difficile* to identify a core set of 404 essential genes and 798 genes that are likely to impact spore production (Dembek *et al*. 2015).

Here, we reported the development of a xylose inducible *mariner*-based transposon system and its exemplification in wild-type *C. acetobutylicum* ATCC 824. Moreover, we described the isolation of a sporulation mutant that contains an insertion within the gene *spo0A* from 200 mutants generated by this transposon system.

## MATERIALS AND METHODS

### Bacterial strains and media

Bacterial strains utilized in this study are listed in Table 1. *Escherichia coli* strains were grown in Luria-Bertani medium at 37°C. *Clostridium* spp. were cultured under anaerobic condition in an anaerobic cabinet (MG1000 Anaerobic Work Station, Don Whitley Scientific Ltd) containing an atmosphere of 80% nitrogen, 10% hydrogen and 10% carbon dioxide. Antibiotics were used at the following concentrations: erythromycin (Em), 20 μg ml^−1^, thiamphenicol (Tm), 15 μg ml^−1^ for *Clostridium* spp.; Em, 500 μg ml^−1^, chloramphenicol (Cm), 25 μg ml^−1^, tetracycline (Tc), 10 μg ml^−1^ for *E. coli*. *Clostridium acetobutylicum* ATCC 824 was grown in *Clostridium* Growth Medium (CGM) (Hartmanis and Gatenbeck 1984) for routine manipulations, or P2 medium (Baer, Blaschek and Smith 1987) with 20 g l^−1^ glucose for auxotrophic mutant screening.

**Table 1.** Bacterial strains and plasmids.

Strains or plasmids	Relevant characteristics^a^	Reference or source^b^
Strains		
*C. acetobutylicum* ATCC 824	Wild type	ATCC
*E.coli* ER2275	*hsdR mcr recA1 endA1*	NEB
*E.coli* DH5α	General cloning host strain	Takara
Plasmids		
pAN1	Φ3TI, p15a origin, Spe^r^	Mermelstein and Papoutsakis (1993)
pMTL82254	*Clostridium* modular plasmid with *catP* reporter, pBP1 (Gram^+^ origin), ColE1+*tra* (Gram^–^ origin), Em^r^	Heap *et al*. (2009)
pMTL82254-Pfdx	*Clostridium* modular plasmid with *catP* reporter expressed by the *fdx* promoter, pBP1 (Gram^+^ origin), ColE1+*tra* (Gram^–^ origin), Em^r^	Zhang, Grosse-Honebrink and Minton (2015)
pMTL82254-Pcac1339	*Clostridium* modular plasmid with *catP* reporter expressed by promoter of cac1339, pBP1 (Gram^+^ origin), ColE1+*tra* (Gram^–^ origin), Em^r^	This study
pMTL82254-Pcac1344	*Clostridium* modular plasmid with *catP* reporter expressed by promoter of cac1344, pBP1 (Gram^+^ origin), ColE1+*tra* (Gram^–^ origin), Em^r^	This study
pMTL82254-Pcac2612	*Clostridium* modular plasmid with *catP* reporter expressed by promoter of cac2612, pBP1 (Gram^+^ origin), ColE1+*tra* (Gram^–^ origin), Em^r^	This study
pMTL83151	*Clostridium* modular plasmid used for construction of IPTG inducible promoter system, pCB102 (Gram^+^ origin), ColE1+*tra* (Gram^–^ origin), Tm^r^	Heap *et al*. (2009)
pMTL-SC0	Transposon plasmid with pBP1 replicon, Tm^r^	Cartman and Minton (2010)
pMTL-YG0	Derived from pMTL-SC0 by replacing pBP1 with pCB102 replicon	This study
pMTL-YG3	Derived from pMTL-YG0 by introducing the promoter of cac1339 to express the transposase *Himar1* C9	This study

^a^ *hsdR*, host-specific restriction deficient; *mcr*, methylcytosine-specific restriction abolished; *recA1*, homologous recombination abolished; *endA1*, endonucleases abolished; Spe^r^, spectinomycin resistance; Em^r^, erythromycin resistance; Tm^r^: thiamphenicol resistance; pBP1, Gram-positive origin of replication; pCB102, Gram-positive origin of replication, which was unstable in *C. acetobutylicum*

^b^ATCC, American Type Culture Collection; NEB, New England Biolabs.

### Plasmids, primers, DNA techniques

Plasmids and primers used in this study are listed in Tables 1 and 2. Chromosomal DNA preparation, plasmid isolation and purification of DNA fragments from agarose gels were carried out using the DNeasy Tissue kit, the QIAprep Miniprep kit and the QIAquick Gel Extraction kit, respectively (Qiagen, Manchester, UK). Restriction enzymes were supplied by New England Biolabs and were used according to the manufacturer's instructions. *Escherichia coli* strains were transformed by electroporation using a Gene-Pulser (Bio-Rad), as recommended by the manufacturer. PCR amplifications were carried out using the KOD Hot Start Master Mix (Merck, Darmstadt, Germany). Oligonucleotides used in this study are detailed in Table 2, which were synthesized by Eurofins MWG Operon, Germany.

**Table 2.** Oligonucleotide primers used in this study.

Primer name	Sequence (5′-3′)	Description
1339-F1	CATCATATGGAAAACTCCTCCTTAAGATTTATAT	Amplify Cac1339 promoter
1339-R1	ACCGCGGCCGCTTTATATTTAGTCCCTTGCCTTGCC	Amplify Cac1339 promoter
1344-F1	CATCATATGACATTAATAAATTAACTGTTATACT	Amplify Cac1344 promoter
1344-R1	ACCGCGGCCGCTTTTAAAACCCCTTCCCGAAATATT	Amplify Cac1344 promoter
2612-F1	CATCATATGAATCAAACCCCCTTAATTTTAAATA	Amplify Cac2612 promoter
2612-R1	TACCGCGGCCGCAATTATATATTTTATGTGTAGAAT	Amplify Cac2612 promoter
pCB102-F	TCCGGCGCGCCGATAATTTACAGAAAAGAAAATTA	Amplify pCB102 replicon
pCB102-R	TTCGGCCGGCCTGCAGCACATTAAGTATATACTATT	Amplify pCB102 replicon
catP-INV-F1	TATTGTATAGCTTGGTATCATCTCATCATATATCCCCAATTCACC	For inverse PCR
catP-INV-R1	TATTTGTGTGATATCCACTTTAACGGTCATGCTGTAGGTACAAGG	For inverse PCR
catP-Sou-F1	GATTGTTTCCATACCGTTGC	For southern probe synthesis
catP-Sou-R1	AGTTATTAAGTCGGGAGTGC	For southern probe synthesis

To confirm whether transposition had occurred, inverse (INV) PCR was performed according to the previously reported procedure (Cartman and Minton 2010). All DNA Sanger sequencing was carried out by Source BioScience, UK.

To identify the genomic location of transposon insertions, sequence data were analyzed using DNASTAR (www.dnastar.com) and compared to the published genome sequences of *C. acetobutylicum* ATCC 824 (Refseq number NC_003030.1 and NC_001988.2; GenBank accession number AE001437 and AE001438) using Artemis (www.sanger.ac.uk/resources/software/artemis/).

### Plasmid transfer in *C. acetobutylicum*

*Clostridium acetobutylicum* was transformed as described previously (Mermelstein *et al*. 1992). Prior to transformation, plasmid DNA was purified from *E. coli* ER2275 cells containing plasmid pAN1. This plasmid contains the φ3TI methyltransferase gene of *Bacillus subtilis* phage φ3tI, which protects DNA from *Cac*824I restriction activity in *C. acetobutylicum* (Mermelstein and Papoutsakis 1993).

### Construction of plasmids

The promoters of CAC1339 (putative sugar-proton symporter, *araE*), CAC1344 (sugar kinase, possible xylulose kinase, *xylB*) and CAC2612 (xylulose kinase, *xylB*) were amplified using primers 1339-F1/1339-R1, 1344-F1/1344-R1 and 2612-F1/2612-R1 and then cloned into plasmid pMTL82254 via *Nde*I/*Not*I. The final plasmids were sequence verified and named pMTL82254-Pcac1339, pMTL82254-Pcac1344 and pMTL82254-Pcac2612 accordingly.

To swap the existing Gram-positive pBP1 replicon in plasmid pMTL-SC0 (Cartman and Minton 2010) with pCB102, a 2403 bp *Asc*I/*Fse*I fragment was excised from pMTL-SC0 and then replaced with a 1625 bp *Asc*I/*Fse*I fragment from pMTL83151, resulting plasmid pMTL-YG0. To generate the final transposon plasmid, the 269 bp *Not*I-*Nde*I fragment encompassing the promoter of the CAC1339 gene was cloned into plasmid pMTL-YG0 to express *Himar1* C9 transposase, thus, giving rise to plasmid pMTL-YG3 (Fig. 2).

### Chloramphenicol acetyltransferase assay

Chloramphenicol acetyltransferase (CAT) activity was determined according to the method of Shaw (1975). A quartz cuvette was prepared containing 540 μl of 100 mM Tris buffer (pH 7.8), 200 μl of 2.5 mM DTNB (5,5′-dithiobis-2-nitrobenzoic acid) solution in 100 mM Tris buffer (pH 7.8), 200 μl of 5.0 mM freshly prepared acetyl Coenzyme A solution in deionized water and 10 μl of cell lysate. The cuvette was pre-warmed to 25°C, and the reaction initiated by adding 10 μl of 0.3% w/v Cm solution in deionized water. The initial rate of increase of absorption at 412 nm was measured using an Analytik Jena SPECORD^®^ 250 PLUS spectrophotometer.

### Isolation of transposon mutants

The *mariner* transposon plasmid pMTL-YG3 was *in vivo* methylated and transformed into *C. acetobutylicum* ATCC 824 by electroporation as described (Heap *et al*. 2009), and transformants were selected on CGM agar plates with 2% glucose (Hartmanis and Gatenbeck 1984), supplemented with 20 μg ml^−1^ erythromycin, for 48 h. All transformants were harvested by flushing the whole plate with CGM broth, then spread onto CGM agar plates with 2% xylose supplemented with 15 μg ml^−1^ thiamphenicol, to induce *Himar1* C9 transposase expression and select transposon mutants. After 48 h of induction, all growth on agar plates were harvested by flushing the whole plate with CGM broth. For further characterization, glycerol was added to the final concentration of 10% and stored at −80°C.

To examine the transposon insertion rate and eliminate plasmids, the harvested cell was subcultured into liquid CGM medium using glucose as the sole carbon source, for three passages with 5% inoculum (12 h every passage). And serial dilutions were made and plated onto CGM agars in triplicate: CGM agar plates without any antibiotics, CGM agar plates supplemented with thiamphenicol and CGM agar plates supplemented with erythromycin.

### INV PCR and DNA sequence analysis

INV PCRs were performed as described previously (Cartman and Minton 2010). Briefly, genomic DNA was isolated from mutants and digested overnight with *Hin*dIII (200 ng μl^−1^). The *Hin*dIII restriction endonuclease was heat inactivated at 65°C for 30 min. The DNA was diluted to 5 ng μl^−1^, and then T4 DNA ligase was added to favor self-ligation of the DNA fragments. Ligation reaction was performed at 16°C overnight. Then, the T4 ligase was heat inactivated (65°C for 30 min). INV PCRs were carried out in 50 μl volumes using primers catP-INV-F1 and catP-INV-R1.

### Southern blot

Southern blot analysis was carried out using a DIG (digoxigenin) High Prime DNA Labeling and Detection Starter kit I (Roche) as instructed by the manufacturer.

## RESULTS AND DISCUSSION

### Identification of a xylose inducible promoter

**Figure 1.**
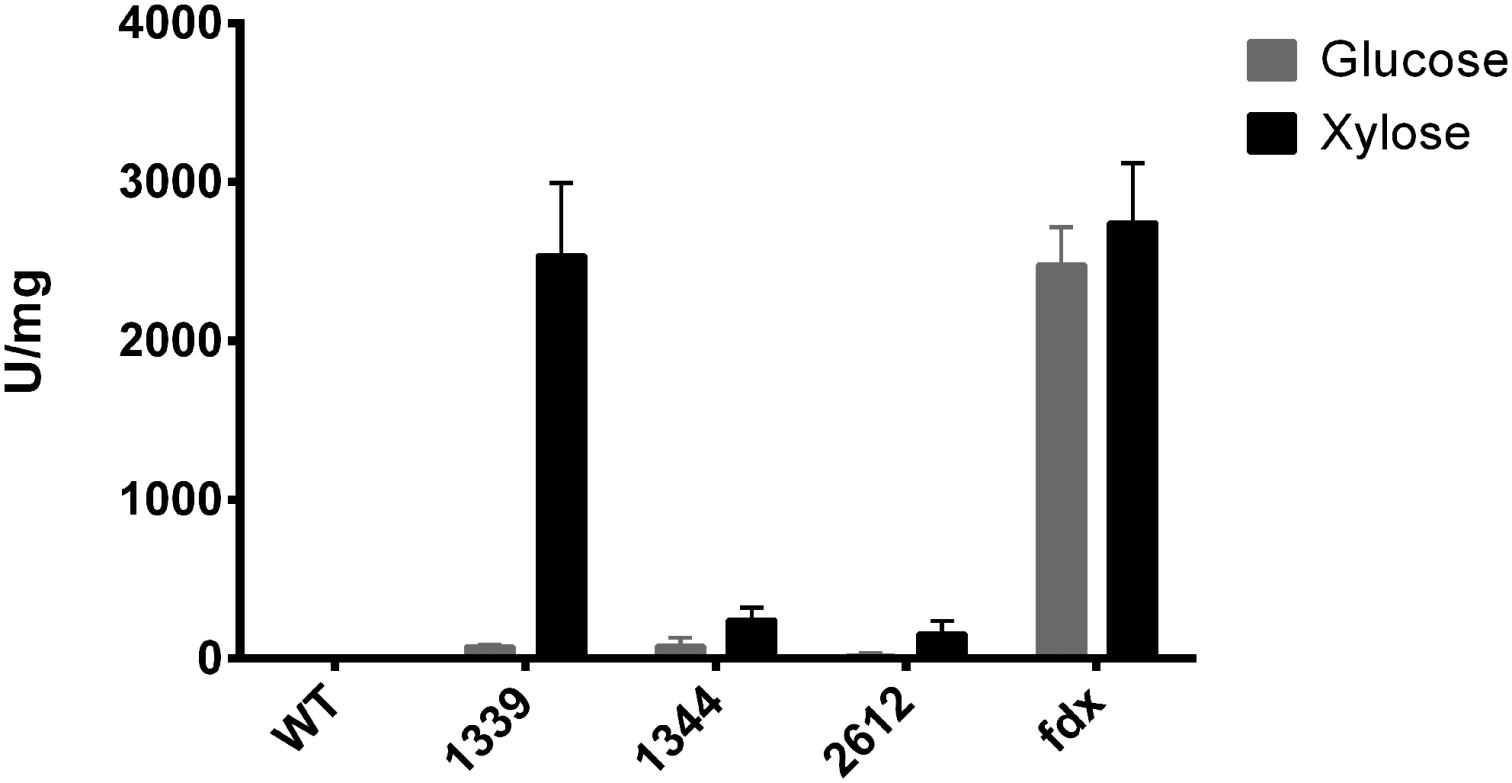
CAT activities showing the strength of the promoters of the CAC1339, CAC1344, CAC2612 genes of *C. acetobutylicum* compared to the promoter of the *fdx* gene of *C. pasteurianum* in the presence of either glucose (grey) or xylose (black) as the sole carbon source. Data are representative of three replicates.

Potential xylose inducible promoters were identified from DNA microarray data generated during growth of *C. acetobutylicum* ATCC 824 on a mixture of glucose and xylose (Grimmler *et al*. 2010). In the described experiments, RNA samples were taken from cells grown in a phosphate-limited continuous culture in which either glucose or xylose was the sole carbon source. By comparing the expression profiles of the two cultures, it was possible to identify three genes that were significantly up regulated when growing on xylose compared to glucose. These were CAC1339 (Putative sugar-proton symporter, *araE*), CAC1344 (Sugar kinase, possible xylulose kinase, *xylB*) and CAC2612 (Xylulose kinase, *xylB*). Accordingly, the DNA fragments encompassing the promoter regions of these three genes were cloned upstream of the promoter-less *catP* gene of pMTL82254 to generate the plasmids pMTL82254-Pcac1339, pMTL82254-Pcac1344 and pMTL82254-Pcac2612. Each plasmid was transformed into *C. acetobutylicum* as described, along with the positive control of plasmid pMTL82254-Pfdx (*catP* reporter gene expressed from the promoter of the *C. pasteurianum* ferredoxin gene, (Zhang, Grosse-Honebrink and Minton 2015) and the negative control of plasmid pMTL82254 that contained a *catP* reporter gene without a promoter (Heap *et al*. 2009). The erythromycin resistant transformants obtained were purified by restreaking on CGM agar supplemented with erythromycin and then used in comparative experiments, growing either on glucose or xylose, and measuring production of CAT. Briefly, cells carrying each of the five plasmids were grown up overnight in 5 ml of CGM broth containing erythromycin (20 μg ml^−1^) and a 1.5 ml aliquot was used to inoculate two batches of 30 ml of P2 medium (Baer, Blaschek and Smith 1987) containing either 5% (w/v) glucose or 5% (w/v) xylose as the carbon source. The cultures were then incubated until their OD_600_ reached 1.1, at which point (7 h for glucose grown cells and 17 h for xylose grown cells) the cells were harvested by centrifugation (13 000 rpm at 4°C for 10 min) and the pellets obtained were stored at −80°C. Lysates were then prepared and the levels of CAT present were determined by enzyme assay (as described in Materials and Methods). The data demonstrated (Fig. 1) that the cells carrying the plasmid (pMTL82254-Pcac1339) which incorporated the promoter of CAC1339 (Pcac1339) showed the highest CAT activity in the presence of xylose with a 33-fold increase compared to cells grown on glucose. This xylose-induced promoter activity is almost equivalent to the *fdx* promoter (Pfdx), one of the strongest promoters in *Clostridium* (Takamizawa *et al*. 2004). Production of CAT by cells carrying plasmids which containing the other two promoters was also induced when growing on xylose compared to glucose. However, the level of CAT activity in cells in which *catP* was under the transcriptional control of the Pcac1344 promoter was only one-tenth of that with the Pfdx promoter, while the Pcac2612 activity was apparently even lower (Fig. 1). Given the level of xylose induction achieved, and the relative promoter strength, it was concluded that the promoter of CAC1339 (*araE*) was the most appropriate transcriptional system for the controlled expression of the *Himar1* C9 transposase gene expression in the planned transposon mutagenesis system.

### Construction of a xylose inducible *mariner*-based transposon system for *C. acetobutylicum*

**Figure 2.**
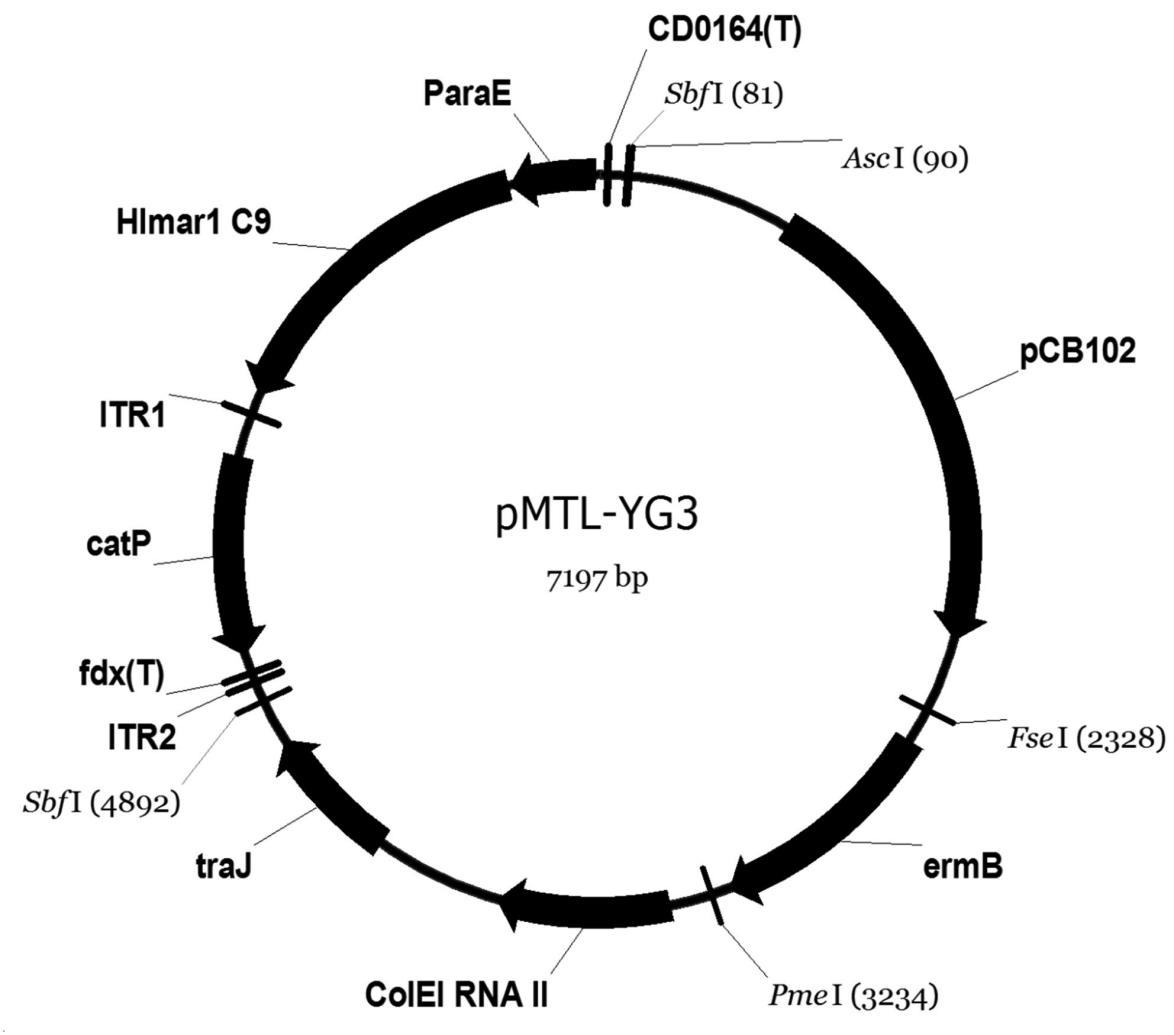
Vector map of plasmid pMTL-YG3. Expression of the hyperactive *mariner* transposase gene *Himar1* C9 was under the control of the P*_araE_* promoter of the *C. acetobutylicum* CAC1339 gene (possible sugar-proton symporter, *araE*). The plasmid backbone consisted of the pCB102 replicon of *C. butyricum* (Minton and Morris 1981), the macrolide-lincosamide-streptogramin B antibiotic resistance gene *ermB*, the Gram-negative replicon ColE1, and the conjugal transfer function *traJ*. The whole *mariner* element (i.e. transposase gene and *catP* mini-transposon) can be excised as an *Sbf*I fragment. The transcriptional terminators (T) are identical in sequence to those found immediately downstream of the *fdx* gene of *C. pasteurianum* and the CD0164 open reading frame of *C. difficile* 630. This vector conforms to the pMTL80000 modular system for *Clostridium* shuttle plasmids (Heap *et al*. 2009).

To construct the transposon delivery vector, an appropriate *Clostridium* replicon needs to be selected to facilitate rapid plasmid loss after the transposition events. Segregational stability studies showed that the replicon of pCB102 is the most segregationally unstable in *C. acetobutylicum* ATCC 824, suggesting that it can be used as a ‘pseudo-suicide vector’ (Cartman and Minton 2010) in the envisaged xylose inducible transposon system. After swapping the existing Gram-positive pBP1 replicon in plasmid pMTL-SC0 (Cartman and Minton 2010) with pCB102, and the cloning of the Pcac1339 promoter proximal to the *Himar1* C9 transposase gene, the desired xylose inducible *mariner* transposon plasmid was generated and designated pMTL-YG3 (Fig. 2).

### Isolation and analysis of transposon mutants

To test its effectiveness as a transposon delivery system, the plasmid pMTL-YG3 was transformed into *C. acetobutylicum* and transformants were selected by plating on CGM agar plates containing glucose as the sole carbon source and supplemented with erythromycin. After incubated at 37°C for 48 h, all transformant colonies were harvested from the agar surface and resuspended in 6 ml of CGM broth and replated as 30 × 0.2 ml aliquots on 30 individual CGM agar plates containing xylose as the sole carbon source. In this case, the agar media was also supplemented with thiamphenicol to select for transposon events. After 48 h, approximately 1000 colonies were visible on each agar plate. All of the colonies were scraped from each of the 30 plates (roughly 30 000) and resuspended in CGM broth containing 10% (v/v) glycerol for storage purposes. To eliminate the transposon delivery vector, the harvested pools of transposon mutants were passaged a total of three times through liquid CGM medium containing glucose as the sole carbon source but lacking any antibiotic supplementation (see Materials and Methods). Serial dilutions of the cell suspension obtained were plated onto CGM agar plates in triplicate. To determine the transposon insertion rate and percentage of plasmid loss, CFU ml^−1^ were estimated on CGM agar plates containing no supplementation or agar media supplemented with either thiamphenicol (Tm) or erythromycin (Em). After 48 h of incubation, 358, 329 and 7 colonies were visible on the agar plate without any antibiotics, supplemented with Tm or Em, respectively. These data demonstrated over 90% of the total colonies contained a transposon insertion, and only a small fraction (about 2%) of those still harbored the transposon delivery vehicle at the final plating stage. A total of 69 colonies from plates supplemented with Tm were picked and patch plated onto appropriate solidified rich media containing either Tm or Em to confirm the percentage of cells that had lost the plasmid. All 69 colonies were Tm resistant and Em sensitive, indicating that they carry transposon insertions in the chromosome and not the transposon delivery plasmid.

**Figure 3.**
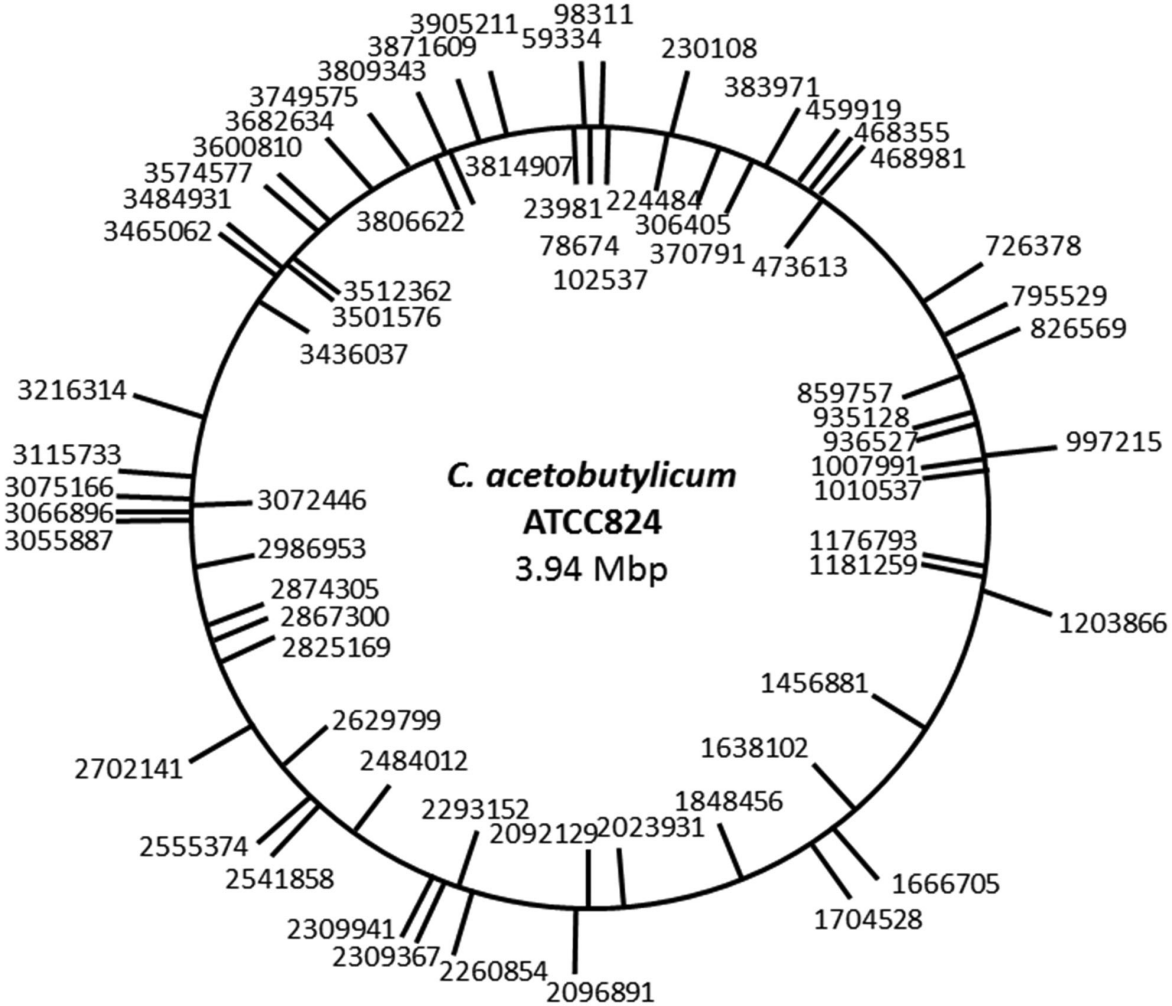
Genetic map of *mariner* transposon insertions. A total of 69 independent transposon insertions were sequenced. Insertions in the plus orientation are marked on the circle exterior. Insertions in the minus orientation are marked on the circle interior. Numbers indicate the precise point of insertion according to genome sequence data for *C. acetobutylicum* ATCC 824 (NCBI Ref Seq number NC_ 003030.1; GenBank accession number AE001437) (Nolling *et al*. 2001).

To determine the insertion sites and establish the randomness of the system, genomic DNA was isolated from the above 69 clones and subjected to INV PCR as described (Cartman and Minton 2010). Nucleotide sequencing of the amplified DNA fragments demonstrated that all 69 clones had a single transposon insertion distributed at random around the genome (Fig. 3), indicating that there was no preferred target site within the genome of *C. acetobutylicum* ATCC 824. Overall, there were 36 insertions in the plus strand and 33 in the minus strand. Moreover, according to the sequencing results, 58 of the 69 insertions sequenced (84%) were located within encoding regions.

**Figure 4.**
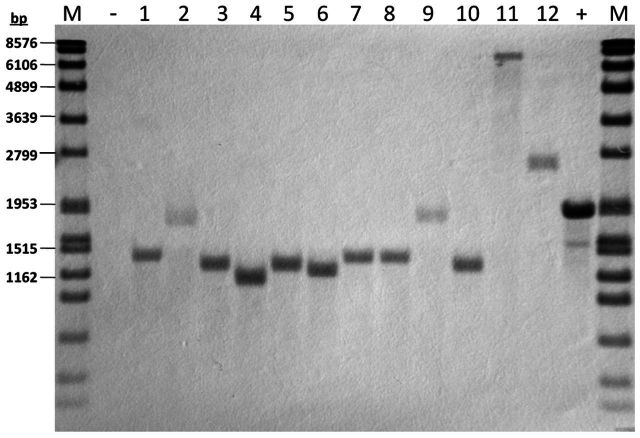
Southern blot analysis of *mariner* transposon insertions in the genome of *C. acetobutylicum*. M: marker; ‘−’: wild-type strain; ‘+’: plasmid pMTL-YG3. Lane 1–12: colonies harboring transposon insertions.

To further examine the independence of each transposition event, Southern blot analysis was performed, in which 12 Tm^r^ colonies from above mentioned mutants library, a wild-type strain (negative control) and the plasmid pMTL-YG3 (positive control) were adopted. As shown in Fig. 4, all 12 colonies had a single transposon insertion, thus, indicating that all transposition events here were a single transposon insertion in the genome.

### Phenotype screens and identification of transposon insertions

To demonstrate that the developed xylose inducible *mariner*-based transposon system can be used for the isolation of mutants with a desired phenotype, over 200 mutants were tested for defects in germination/sporulation. Briefly, the mutant library was plated onto CGM agar plates (supplemented with Tm). When colonies were visible on the plates after incubation at 37°C for 48–72 h, replica cultures of over 200 mutant colonies were inoculated into fresh CBM liquid broth (O'Brien and Morris 1971) in 96-well microtitre plates and anaerobically incubated for two weeks to form spores. The cultures in one 96-well microtitre plate were subjected to heat treatment (80°C for 10 min) and then plated onto CGM agar plate supplemented with Tm, while the cultures in the replicated 96-well microtitre plate were directly plated onto CGM agar plate. Fortunately, we obtained a mutant which was defective in its ability to form colonies after heat shock but that was still able to generate colonies in the absence of a heat treatment. By sequencing the INV PCR products of this *spo*^_^ mutant, we were able to show that it contained an insertion in the *spo0A* gene. This gene encodes the master regulator of sporulation, Spo0A, and has been found to be essential for both sporulation and solvent production in *C. acetobutylicum* (Harris, Welker and Papoutsakis 2002). Although no novel genes affecting sporulation were identified, a consequence of the low number of mutants screened, the successful isolation of the *spo0A* mutant did demonstrate that in principle the transposon mutagenesis system developed can be used for forward genetic studies.

In summary, we have constructed a novel random mutagenesis system in which the production of the *mariner* transposase, and thereby transposition events, is controlled through the use of a xylose inducible promoter. Induction is, therefore, simply controlled through the temporal presence of xylose in the agar medium employed. The use of an inducible promoter to control transposase production may overcome the drawbacks of using constitutive promoters, i.e. early-stage transposition events immediately after electro-transformation which would result in a lack of randomness of the mutant library.
